# Large Direct Repeats Flank Genomic Rearrangements between a New Clinical Isolate of *Francisella tularensis* subsp. *tularensis* A1 and Schu S4

**DOI:** 10.1371/journal.pone.0009007

**Published:** 2010-02-03

**Authors:** Ufuk Nalbantoglu, Khalid Sayood, Michael P. Dempsey, Peter C. Iwen, Stephen C. Francesconi, Ravi D. Barabote, Gary Xie, Thomas S. Brettin, Steven H. Hinrichs, Paul D. Fey

**Affiliations:** 1 Department of Pathology and Microbiology, University of Nebraska Medical Center, Omaha, Nebraska, United States of America; 2 Department of Electrical Engineering, University of Nebraska-Lincoln, Lincoln, Nebraska, United States of America; 3 Bioscience Division, Los Alamos National Laboratory, Los Alamos, New Mexico, United States of America; 4 DOE Joint Genome Institute, Walnut Creek, California, United States of America; 5 Division of Microbiology, Armed Forces Institute of Pathology, Washington, D.C., United States of America; 6 Naval Medical Research Center, Silver Spring, Maryland, United States of America; 7 Oak Ridge National Laboratory, Oak Ridge, Tennessee, United States of America; University of Hyderabad, India

## Abstract

*Francisella tularensis* subspecies *tularensis* consists of two separate populations A1 and A2. This report describes the complete genome sequence of NE061598, an *F. tularensis* subspecies *tularensis* A1 isolated in 1998 from a human with clinical disease in Nebraska, United States of America. The genome sequence was compared to Schu S4, an *F. tularensis* subspecies *tularensis* A1a strain originally isolated in Ohio in 1941. It was determined that there were 25 nucleotide polymorphisms (22 SNPs and 3 indels) between Schu S4 and NE061598; two of these polymorphisms were in potential virulence loci. Pulsed-field gel electrophoresis analysis demonstrated that NE061598 was an A1a genotype. Other differences included repeat sequences (n = 11 separate loci), four of which were contained in coding sequences, and an inversion and rearrangement probably mediated by insertion sequences and the previously identified direct repeats I, II, and III. Five new variable-number tandem repeats were identified; three of these five were unique in NE061598 compared to Schu S4. Importantly, there was no gene loss or gain identified between NE061598 and Schu S4. Interpretation of these data suggests there is significant sequence conservation and chromosomal synteny within the A1 population. Further studies are needed to determine the biological properties driving the selective pressure that maintains the chromosomal structure of this monomorphic pathogen.

## Introduction

*Francisella tularensis* is a highly pathogenic gram-negative cocco-bacillus that is the causative agent of tularemia, commonly referred to as “rabbit fever.” The large majority of disease is ulceroglandular in nature and can be traced to contact with an infected host (e.g. rabbit or cat) or vector (e.g. tick or mosquito); however more serious forms of disease such as pneumonic tularemia can be life-threatening, and therefore *F. tularensis* is considered a potential biowarfare agent. There are three recognized subspecies of *F. tularensis* including *tularensis* (commonly referred to as type A), *holarctica* (commonly referred to as type B), and *mediasiatica* as well as a closely related species *F. novicida*. These subspecies are associated with important geographic differences in their distribution with *F. tularensis holarctica* found throughout the northern temperate regions of both hemispheres whereas subspecies *tularensis* is found primarily in North America. In addition, the population of *F. tularensis* subspecies *tularensis* consists of two major, geographically isolated clades, A1 and A2 [1], [2]. The A2 population has been isolated in the western United States whereas the A1 population is found east of the Rocky Mountains, primarily in the Ozark mountain regions of Missouri, Oklahoma and Arkansas. The genomes of two *F. tularensis* subspecies *tularensis* A1 isolates (Schu S4 and FSC198) have recently been sequenced; FSC198 was isolated from Slovakia in 1986 whereas Schu S4, an often-utilized virulent laboratory strain, is a clinical isolate obtained from Ohio in 1941 [3], [4]. In addition, a draft sequence of a separate *F. tularensis* subsp. *tularensis* A.I isolate, FSC033, was also recently published [5]. FSC033 was isolated from a squirrel in Georgia, USA. Genomic comparisons between FSC198 and Schu S4 revealed remarkable sequence conservation; only 8 SNP and three variable number tandem repeat (VNTR) differences were noted [3]. Chaudhri et al. [3] have suggested that the close similarity between FSC198 and Schu S4 indicated that the FSC198 strain may have derived from Schu S4. Preliminary analysis between a recent human clinical isolate of *F. tularensis* subsp. *tularensis* obtained in 1998 in Nebraska and Schu S4 revealed distinguishing characteristics [6]. This presented an opportunity to further examine the genomic diversity within the A1 population, and therefore, the complete sequence of a *F. tularensis* subspecies *tularensis* A1 isolate NE061598 was determined. The genomes of the four A1 isolates that have been fully or partially sequenced (SchuS4, FSC198, NE061598 and FSC033) were compared in light of their temporal and spatial separation. This analysis demonstrated that the *F. tularensis* subsp. *tularensis* A1 population, as represented by these isolates, is highly clonal and displays a high degree of DNA sequence conservation and chromosomal synteny. The primary chromosomal differences between NE061598 and Schu S4/FSC198/FSC033 were due to rearrangements occurring between large direct repeats and insertion sequences.

## Results

### General Features

The genomic sequence *of Francisella tularensis* subsp. *tularensis* NE061598 (GenBank accession number CP001633 or at http://bioinfo.unl.edu/NE061598genome) consists of a single circular chromosome of size 1,892,681 base pairs (bp). General characteristics of the NE061598 genome are shown in Table 1. Using pulsed-field gel electrophoresis, Kugeler et al have demonstrated the population of *F. tularensis* subsp. *tularensis* A.I can be divided into at least two separate groups, A1a and A1b [2]. Previous PFGE analysis of NE061598 using both *Pme*I and *Bam*HI suggested that it was a subtype A1a (data not shown and [6]).

**Table 1:** Genomic characteristics of *F. tularensis* subsp. *tularensis* NE061598.

Length (bp)	1892681
GC Content (%)	32.26
Total Genes	1850
Protein Coding Genes	1601
Genes Assigned Function	1185
Hypothetical proteins	416
Disrupted ORFs	201
Large Duplicated Regions	2
Transposons (IS elements)	75
tRNA	38
rRNA	10
sRNA	2
Average Gene Length (nt)	1068
Percent Coding	90.40%

### Comparison to the Other Type A1 Strains

The NE061598 genome sequence contains 65 bp more than the FSC198 sequence [3] and 94 bp less than the Schu S4 sequence [4]. Previous bioinformatic analysis of the FSC198 and Schu S4 genomes demonstrated that there were only eight single nucleotide polymorphisms (SNPs) and three VNTR differences between these two isolates [3]. Therefore, based on the known genomic similarity between Schu S4 and FSC198, NE061598 was compared with Schu S4 (Genbank accession number AJ749949 and the Refseq accession no. NC_006570). The regions of difference between Schu S4 and NE061598 were divided into 2 types: small tandem repeats (Table 2) and rearrangements (Table 3). The VNTR's listed in Table 2 accounted for the difference in size between the two isolates. Table 2 consists of known VNTR markers used previously for MLVA analysis [6], [7] in addition to five newly identified tandem repeat differences (VNTR 1–5) discovered between NE061598 and Schu S4. Only one of the five new VNTRs was found within an open reading frame.

**Table 2:** VNTR markers and their differences between Schu S4 and NE061598.

VNTR^a^ Marker	Repeat motif	Repeat size (nt)^b^	Genomic Location	Repeat copy no., strain SCHU S4	Repeat copy no., strain NE061598
Ft-M1	AAT	3	I (−76)	3	3
Ft-M2	TAAATA	6	G (+12)	4	5
Ft-M3	AATAAGGAT	9	G (+1401)	25	20
Ft-M4	TTGTT	5	G (+55)	3	3
Ft-M5	TTTCTACAAATATCTT	16	I (−21)	3	2
Ft-M6	TTGGTGAACTTTCTTGCTCTT	21	G (+1160)	4	5
Ft-M7	TTTCTACAAATATCTT	16	I (−21)	4	4
Ft-M8	TTTCTACAAATATCTT	16	I (−21)	4	4
Ft-M9	TTTCTACAAATATCTT	16	I (−21)	4	9
Ft-M10	TTTCTACAAATATCTT	16	I (−21)	18	8
Ft-M11	AATTATAAAT	10	I (−113)	5	5
Ft-M12	TAGCTTTTTT	10	I (−113)	2	2
Ft-M13	CTCCAGGACCAA	12	G (+1174)	2	2
Ft-M14	TCATTA	6	G (+67)	3	3
Ft-M15	ATACTT	6	G (+32)	2	2
Ft-M16	TAAAAGTAAG	10	I (+551)	2	2
Ft-M17	TATTTA	6	G (+484)	3	3
Ft-M18	CATTAA	6	I (−52)	4	4
Ft-M19	TAAATTTCTCATA	13	I (−20)	2	2
Ft-M20	ATTATTTTGATC	12	G (+1964)	3	3
Ft-M21	TCAATTA	7	G (+586)	3	4
Ft-M22	AAAAAT	6	G (+2254)	2	2
Ft-M23	AAGTAGCATTGTCACGACCTCCT	23	I (+1864)	2	2
Ft-M24	ATAAATTATTTATTTTGATTA	21	I (−93)	1	1
Ft-M25	GT	2	G (+525)	5	5
VNTR-1	CAAAGACA	8	I (−392)	1	3
VNTR-2	TTTATATAAGT	11	I (−42)	3	2
VNTR-3	GAAAATAA	8	G (+282)	1	2
VNTR-4	TTCTACAAATATCTTT	16	I (+22)	2	3
VNTR-5	AAAATGCCATCATATAGCCAAGATTTTAG	29	I (−32)	1	1

a FtM1-FtM25 VNTR markers as previously reported by Johansson et al. [7]. New VTNR polymorphisms identified in this study are listed as VNTR1 through VNTR-5.

b Indicates repeat size in nucleotides.

c “G” indicates that the repeat is located within an open reading frame (genic) whereas “I” indicates that the repeat is located within an intergenic region. Distance to predicted translation start site is indicated in nucleotides. “+” or “−” indicates that the translation start site is downstream or upstream of repeat motif, respectively (as reported by Johansson et al. [7]).

Compared to the published Schu S4 genome sequence, NE061598 had 25 polymorphisms (22 SNPs and 3 indels; Table 4). All SNP and indel differences were confirmed by repeat sequence analysis. Of the 22 confirmed SNPs, 6 were synonomous SNPs, 5 were intergenic SNPs, and 11 were nonsynonomous. There were no SNPs in rRNA or tRNA genes. Petrosino et al. [8] have identified 268 virulence genes associated with *F. tularensis*. Comparing NE061598 to Schu S4, only two of the proposed virulence genes identified by Petrosino et al. [8] were determined to have SNPs. These include a ferrous iron transport protein (FTT0249) and 2-isopropylmalate synthase (FTT0252). Both contain non-synonymous polymorphisms that result in a non-conservative amino acid substitution; it is unknown whether these mutations have any effect on protein function.

Apart from the rearrangements and polymorphisms, the main reason for the remaining genomic differences in composition and length between NE061598 and Schu S4 were found to be due to differences in the VNTR's. VNTR analysis has been very useful in epidemiological and population analyses of *Francisella* [6], [7]. Of the twelve tandem repeats that have a unique number of repeats in NE061598 in comparison to Schu S4, 7 (FtM5, FtM9, FtM10, FtM21, VNTR-1, VNTR-2, and VNTR-4) occur in intergenic regions, and the remaining 4 (FtM2, FtM3, FtM6, and VNTR-3) are in coding regions (Table 3). Of these four, one repeat in the gene for a hypothetical protein (FtM2; FTT1800c [Schu S4] and NE6158_10490 [NE061598]) inserted two amino acids into the translated sequence. Another repeat in a gene for a hypothetical protein (VNTR3; FTT0877c [Schu S4]) resulted in a premature stop codon in NE061598. An insertion of 7 amino acids was observed in an ATP-dependent DNA helicase protein in NE061598 compared to Schu S4 (FTT1395c [Schu S4] and NE61598_07740 [NE061598]). Lastly, one tandem repeat difference (FtM3) appeared to eliminate a premature stop codon in a pseudogene in Schu S4 (TPR repeat region protein; FTT0294 [Schu S4] and NE61598_0160 [NE061598]). This difference resulted in a deletion of the repeat NKDNKDNKD. Importantly, NE061598 does not encode any unique genes that are not found in Schu S4.

**Table 3:** Description of six local collinear blocks (LCBs) between NE061598 and Schu S4.

LCB	Type	NE061598 Position	Schu S4 position
1	Conserved	1-352156	1-352087
2	Inversion	352157-381876	381807-352088
3	Conserved	381877-1312701	381808-1312781
4	Rearrangement	1312702-1700690	1379901-1767877
5	Rearrangement	1700691-1767602	1307424-1374335
6	Conserved	1767603-1892681	1767671-1892775

**Table 4:** Non-synonymous SNPs, synonymous SNPs, and indels discovered between NE061598 and Schu S4.

Schu S4/NE061598^a^		Nucleotide change^b^	Type^c^	ORF_ID^d^	Product^e^	Putative amino acid change^f^
157940	158036	A/C	sSNP	FTT0144	DNA-directed RNA polymerase subunit beta	SYN
218776	218872	G/A	iSNP	IGS	intergenic space or other non-protein-coding region	−
262990	263086	C/G	nSNP	FTT0249	ferrous iron transport protein [17]	T/R
269208	269304	C/T	nSNP	FTT0252	2-isopropylmalate synthase	S/F
297337	297433	C/T	sSNP	FTT0282	Cytochrome O ubiquinol oxidase subunit I	SYN
989503	989567	T/–	deletion	IGS	intergenic space or other non-protein-coding region	
1459387	1392208	G/–	deletion	IGS	intergenic space or other non-protein-coding region	
727330	727387	A/G	nSNP	FTT0708	major facilitator superfamily (MFS) transport protein	I/V
753071	753128	G/T	nSNP	FTT0729	ABC transporter, membrane protein	G/W
793639	793696	C/T	sSNP	FTT0773	50S ribosomal protein L27	SYN
853540	853597	C/A	nSNP	FTT0839	hypothetical membrane protein	H/N
920302	920367	G/A	nSNP	FTT0912c	ribosomal large subunit methyltransferase J	L/F
932205	932270	T/C	iSNP	IGS	intergenic space or other non-protein-coding region	–
1154882	1154948	A/T	iSNP	IGS	intergenic space or other non-protein-coding region	–
1223209	1223273	T/C	nSNP	FTT1204c	hypothetical membrane protein	T/A
1296176	1296067	C/T	sSNP	FTT1273	50S ribosomal protein L13	SYN
1351129	1744396	T/C	nSNP	FTT1323	Methylase	L/S
1419877	1352678	C/T	nSNP	FTT1373	3-oxoacyl-[acyl carrier protein] synthase III	P/S
1423162	1355963	A/G	nSNP	FTT1377	3-oxoacyl-[acyl-carrier-protein] synthase II	S/G
1525732	1458553	G/A	sSNP	FTT1473c	Galactose-proton symporter, major facilitator superfamily (MFS) transport protein	SYN
1700620	1633433	C/T	sSNP	FTT1635	cell division protein (post-translational processing & secretion) [18]	SYN
1738053	1670866	T/C	iSNP	IGS	intergenic space or other non-protein-coding region	–
1833651	1833583	T/C	nSNP	FTT1744c	indolepyruvate decarboxylase	Y/C
1540425	1473247	–/A	insertion	IGS	intergenic space or other non-protein-coding region	–
570431	570488	T/C	iSNP	IGS	intergenic space or other non-protein-coding region	–

aNucleotide number at which SNP or indel is located in the Schu S4 and NE061598 genome, respectively.

bPutative nucleotide substitutions or indel in the Schu S4 and NE061598 genomes, respectively, as identified by genomic sequence comparison.

cType of nucleotide substitution. sSNP, synonomous single nucleotide polymorphism; nSNP, non-synonomous single nucleotide polymorphism; iSNP, intergenic single nucleotide polymorphism.

dOpen reading frame (ORF) associated with SNP or indel in the Schu S4 genome sequence. IGS, intergenic sequence.

ePutative protein function of associated ORF.

fAmino acid change of associated SNP or indel.

### Chromosomal Rearrangements

**Figure 1:**
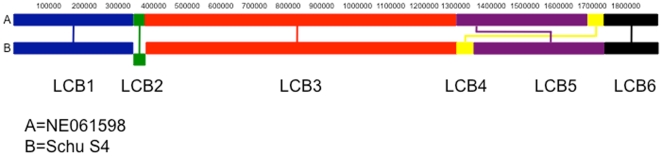
Genome rearrangement representation for NE061598 and Schu S4 genomes. Each local collinear blocks (LCB) 1-6 is represented by a different color. Upside-down blocks (i.e. LCB2) represent the location of the reverse strand, which means an inversion has occurred. Note the rearrangements of LCB4 and LCB5.

In order to describe the chromosomal rearrangements between NE016598 and Schu S4, the genomes were divided into six local collinear blocks (LCBs) as shown in Table 3 and Figure 1. The initial division was performed using the genome rearrangement analysis tool SPRING (Sorting Permutation by Reversals and block-INterchanGes) [9]. These analyses demonstrated that the first, third and sixth LCBs are conserved whereas the second LCB is inverted in NE061598 with respect to Schu S4. The fourth and fifth LCBs are rearranged (Table 3 and Figure 1). These data are consistent with a previous comparison of two type A strains of *Francisella tularensis* subsp. *tularensis*, WY96 (A2) and Schu S4 (A1), which demonstrated the presence of various genome rearrangements due to inversions and block rearrangements mediated by insertion sequences [10]. The remaining LCBs have flanking duplicated regions. Several insertion elements were also observed juxtaposed to the flanking regions of the LCBs (Table 3) that might promote further chromosomal rearrangements during strain divergence. For example, the second LCB is inverted between NE061598 and Schu S4. This inversion is hypothesized to be due to 2969 bp long flanking regions on each side of the inverted region that are reverse complements of each other. These flanking regions are comprised of one ISFtu2 and two additional ISFtu1 insertion sequence elements.

The rearrangements in LCBs four and five are most probably mediated by two large duplicated regions (DR1 and DR2) previously discussed in the genome report comparing WY96 and Schu S4 [10]. These duplicated regions include the *Francisella* Pathogenicity Island (FPI) containing the *iglABCD* operon [11] required for intramacrophage growth. This operon is regulated by the transcription factor MglA that has been shown to regulate a number of virulence factors [12]. These two regions (33,910 bp) occur at locations 1,374,336–1,408,246 (DRI) and 1,767,671–1,801,581 (DRII) in Schu S4. In addition, a 5358 bp segment of the duplicated regions between the 208^th^ and 5565^th^ bases of the duplicated regions, was also duplicated at positions 1,307,425 bp–1,312,781 bp in Schu S4. No structural alterations in the *iglABCD* operon were found in NE061598.

**Figure 2:**
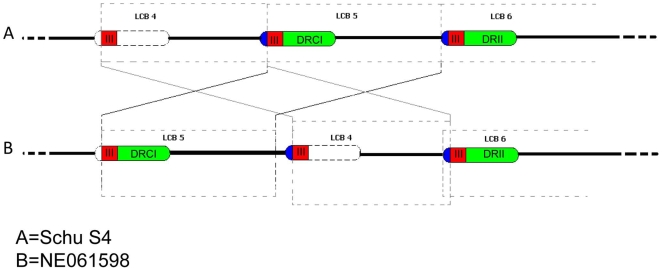
Depiction of genomic rearrangement between local collinear blocks 4 and 5 in NE061598 compared to Schu S4. Direct repeats 1 (DRI) and II (DRII) are colored in green in both 3A (Schu S4) and 3B (NE061598). DRIII, a segment of both DRI and DRII, is colored in red. Note that DRIII is found independently in LCB4. The initial 207 bp of DRI and DRII in Schu S4 is colored in blue. Note that the genomic rearrangement resulted in the loss of this initial 207 bp region in DR1 of NE061598.

**Figure 3:**
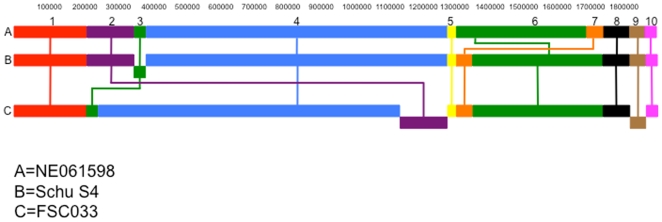
Genome rearrangement representation for NE061598, Schu S4 and FSC033 genomes. Each local collinear blocks (LCB) 1–10 is represented by a different color. Upside-down blocks (i.e. LCBs 3 and 9) represent the location of the reverse strand, which means an inversion has occurred. Each LCB is denoted above NE061598.

The location of DRI and DRII in both Schu S4 and NE061598 are shown in figures 2A and 2B. In addition, DRIII (III, red) is shown which contains the aforementioned 5358 bp long segment of the duplicated regions [10]. Relating these regions to the LCBs noted in Figure 2, DRII is contained in LCB 6 while the other components are contained in LCBs four and five. The rearrangement can be explained as an edit operation in which one block with a partially duplicated flanking region is replaced by another block having DR1 as the flanking region (Figure 3). Consequently, DR2 is conserved in NE061598 but other regions have been transformed to partially duplicated regions. This genomic rearrangement results in the loss of the first 207 bp in DRI of NE061598 (Figure 2). Similar chromosomal changes mediated by these duplicated regions were also observed between Schu S4 and WY96 [10]. WY96 has a conserved copy of DRII and a copy lacking the first 207 bases as in the NE061598 LCB5 region (Figure 3B). These duplicated regions were determined to be the most compositionally different segments of the genome using the Alien Hunter program [13].

While it is known that IS elements are significantly involved in intrachromosomal rearrangement, only one rearrangement associated with insertion sequences was observed when comparing NE061598 to Schu S4. The most parsimonious transformation using the rearrangements and inversions of the collinear blocks involved an inversion of LCB2 and the edit process discussed in Figure 2.

### Comparison of NE061598 and Schu S4 with the Draft Sequence of *F. Tularensis* Subsp. *Tularensis* FSC033

Kugeler et al have demonstrated the population of *F. tularensis* subsp. *tularensis* A1b is associated with higher mortality rates [2]. A prototype A1b isolate, FSC033, has recently been partially sequenced [2], [5]. In order to perform preliminary genomic comparisons between FSC033, NE061598 and Schu S4, the genomes were divided into 10 LCBs as described above (Figure 3). This analysis found that the only major difference between FSC033 and NE061598/Schu S4 was the rearrangement of LCB2 (Figure 3). The genomic organization of FSC033 surrounding DRI and DRII as shown in Figures 1 and 2 was similar to the Schu S4 genomic arrangement. Although few significant differences were observed regarding the genomic synteny between FSC033 (subtype A1b) and NE061598/Schu S4 (subtype A1a), SNP analysis indicated that 123 SNPs and 8 indels were detected between NE061598 and FSC033.

### Transposable Elements

Seven different types (n = 75) of IS elements were found within NE061598 (Table 5). In addition to 50 ISFtu1 elements, NE061598 contains 16 ISFtu2 elements (of which one flanks the inverted LCB 2), 3 ISFtu3 and ISFtu6 elements, and one copy each of ISFtu4, ISFtu5 and ISSod13. All of the insertion sequences found in NE061598 are also present in Schu S4.

**Table 5:** IS element found in NE061598 compared to Schu S4.

IS Elements	Number in NE061598	Number in Schu S4
ISFtu1 (IS630 family)	50	50
ISFtu2	16	16
ISFtu3 (ISNCY family, ISHpal-IS1016)	3	3
ISFtu4 (IS982 family)	1	1
ISFtu5 (IS4 family)	1	1
ISFtu6 (IS1595 family)	3	3
ISSod13	1	1
TOTAL	75	75

## Discussion

Due to the remarkable sequence conservation between Schu S4 and FSC198 [3], speculation was made that these two isolates may have the same origin. Therefore, we proposed to sequence a separate virulent isolate of *F. tularensis* subsp. *tularensis* A1 and compare it with Schu S4 to evaluate the issue of sequence divergence over time. NE061598 was isolated in Nebraska in 1998 from the blood of a patient with ulceroglandular tularemia, Schu S4 was derived in 1941 and FSC198 was isolated in 1986. The availability of a recent clinically virulent isolate of *F. tularensis* subsp. *tularensis* A.I isolate obtained in the mid-western portion of the United States provided the opportunity for an in-depth sequence comparison with other A.I. isolates. Because of the significant temporal separation (45 years) between Schu S4 and NE061598, the sequence conservation between these two isolates was unexpected. Even though VNTR analysis yielded 11 distinct polymorphisms (see Table 2), analysis of the entire genome only yielded 25 additional SNPs/indels. The most significant difference detected was an inversion associated with LCB 2 and rearrangements associated with LCBs 4 and 5 (see Figures 1 and 2); both events were predictably mediated through IS element recombination (LCB 2) or rearrangement mediated by large duplicated regions (LCBs 4 and 5). Significantly, there was no net gain (or loss) of genes within the NE061598 genome in relationship to Schu S4. These data may suggest that the minimal differences observed in pulsed-field RFLP patterns of the *F. tularensis* subsp. *tularensis* A1 population may be due to IS- or direct repeat-mediated rearrangements and is not due to the acquisition of new genes [1], [2], [6]. Furthermore, these data support the notion that this highly monomorphic pathogen [14] may have undergone a recent population bottleneck which may be related to its specific host preference (e.g. lagomorphs, humans) and vectors (e.g. ticks). The further elucidation of the natural reservoir, hosts, and vectors of *F. tularensis* may lead to novel hypotheses of the selective pressure of this A1 population.

Due to the lack of genetic diversity noted within the *F. tularensis* subsp. *tularensis* A1 population, phylogenetic and population structure analyses are problematic and biased especially due to the rapid evolution of VNTR loci and lack of sensitivity of other methodologies [14], [15]. However, whole genome SNP analysis has been successful at probing the population structure of highly monomorphic pathogens such as *B. anthracis* and other highly virulent pathogens [14], [16]. A recent report using a variety of SNP analyses identified 11 subclades within *F. tularensis* subsp. *holarctica* [15]. Phylogenetic analysis suggested that *F. tularensis* subsp. *holarctica* originated from North America and was introduced multiple times into Eurasia. Further studies need to be performed to delineate the complicated population structure of *F. tularensis* subsp. *tularensis* A.I (both A1a and A1b) and its relationship to the *F. tularensis* subsp. *tularensis* A2 population. Data provided in our study may yield canonical SNPs that provide lineage- or strain-specific phylogeny within this subspecies. The utility of these unique SNPs will be evaluated using large repositories of *F. tularensis* subspecies. Lastly, our study suggests that the genomic organization between the A1a and A1b populations may not significantly differ; however, preliminary SNP/indel analysis provides evidence that the increased virulence observed with A1b strains may reside in specific nucleotide alterations and not gene acquisition or loss.

## Materials and Methods

### Genome Sequencing of NE061598

The genome coverage determined at the end of the draft-sequencing phase was 11x and resulted in 19 contigs mapped into 12 scaffolds. The draft phase involved two clone libraries, one small insert library (2200 bp average insert size) and one medium insert library (6289 bp average insert size). Paired end shotgun reads from each of these libraries produced 12218 and 13156 reads respectively. During the finishing phase, seven transposon bomb libraries were created and sequenced to assist with repeat resolution. Four PCR shatter libraries were created and sequenced to assist with hard stops. An additional 528-primer walk reads were created as needed to address low quality regions of the draft assembly. The final genome at the end of the finishing stage was a complete genome with no gaps consisting of 1892901 base pairs. The overall average error rate of the finished genome was less than one error in 100,000 bp. The total number of reads used in the final assembly was 25,531.

### Annotation

The open reading frames of Schu S4 strains were extracted and each ORF was searched for in the NE061598 chromosome using the standard Smith-Waterman algorithm [17]. The hits having accuracy higher than 98% identity were detected as initial annotations. Next, the NCBI annotation pipeline (http://www.ncbi.nlm.nih.gov/genome/guide/build.html) was employed and any missed ORFs were extracted from the output of this pipeline. Eliminating the ORFs and overlapping genes that had already been recognized, protein BLAST searches were performed on filtered predictions of the pipeline.

### Insertion Sequence Element Mapping

Annotated insertion sequence elements that are specific to F. *tularensis* were detected in the NE061598 genome using Smith-Waterman alignment [17].

### SNP Discovery

SNP polymorphisms between Schu S4 and NE061598 were discovered using the *SNPsFinder* program of Los Alamos Laboratories (http://snpsfinder.lanl.gov/UsersManual/index.html). SNP predictions were then curated manually using BLAST (with parameters match: 1 mismatch: −4 existence and extension gaps: −1).

### Genome Rearrangement Discovery

In order to determine the local collinear blocks (LCB), the SPRING tool [7] was utilized. The SPRING parameters for LCB discovery included the following. Block search mode: reversals (inversions) plus block interchange mode; minimum multi-MUM length: 21 bp (closest integer to log_2_ [1892 Kbp], where 1892 is the average genome length); minimum LCB length: 63 bp (3 x minimum multi-MUM); chromosome type: linear. The boundaries of the rearrangements were further optimized using BLAST (expect threshold: 10; word size: 64; match score: 1; mismatch score: −4; existence and extension gaps: −1) around the 10 Kb flanking regions of LCB ends.

### Pulsed-Field Gel Electrophoresis

Agarose embedded DNA was prepared and digested with *Pme*I and *Bam*HI as previously described [18]. RFLP analysis was performed using Bionumerics software (Applied Maths).
